# Supplementary material for: A national survey in United Arab Emirates on practice of passive range of motion by physiotherapists in intensive care unit
Source: PLoS One. 2021 Aug 20;16(8):e0256453. doi: 10.1371/journal.pone.0256453 (PMC8378748; doi:10.1371/journal.pone.0256453)
Supplement: S1 Questionnaire — (DOC) [file pone.0256453.s001.doc]

**A national survey in United Arab Emirates on practice of Mobilization by Physiotherapists in Intensive care units**

**‘The clinical practice of passive limb range of motion by a physiotherapist in ICU’ questionnaire**

## SECTION 1: PHYSIOTHERAPY SERVICE PROVISION

In this section we are seeking information about how physiotherapy is provided in *your* ICU.

## 1. Is there blanket referral for physiotherapy in your ICU? (Blanket referral is when all patients in your ICU are automatically referred for PT.)

□ Yes (please go to question 3) □ No

2. How are patients referred to physiotherapy? Please tick all relevant boxes.

□ Medical staff referral

□ Nursing staff referral

□ Other (please specify)

3. How many full time equivalent (FTE) physiotherapists are allocated to your ICU for your regular

weekday (Sun-Thu) service?

**SECTION 2: PHYSIOTHERAPY ASSESSMENT**

This section pertains to physiotherapy *assessment*, not treatment.

4. Are *all* referred ICU patients *routinely* assessed by a physiotherapist (ie physiotherapist performs a

‘hands on’ assessment)?

□ Yes (please go to question 6) □ No

5. If *all* ICU patients are *not* routinely assessed by a physiotherapist, what do you use to determine

whether a ‘hands on’ physiotherapy assessment is required? Please tick all relevant boxes.

□ Physiotherapist’s judgement of whether physiotherapy is indicated based on a review of each patient’s medical record

□ Current medical status of patient (eg stable or unstable)

□ Other (please specify)

6. Do you *routinely* assess passive limb range of movement (ROM) for *all* patients?

□ Yes (please go to question 8) □ No

7. If you do *not* routinely assess *all* patients’ passive limb ROM, what criteria do you use to determine

whether physiotherapy assessment of patients’ passive limb ROM is required or not? Please tick all relevant boxes.

□ Intubation status

□ Patient sedation

□ ICU length of stay is longer than days (please specify number of days)

□ Reason for admission

□ Past medical history

□ Other (please specify)

□ Never assess passive joint ROM (please go to question 12)

8. When assessing patients’ passive limb ROM, which joints do you *routinely* assess?

□ All joints

□ Selected joints (please specify which joints)

9. How do you measure patients’ passive limb ROM? Please tick all relevant boxes.

□ Visual estimation of joint ROM

□ Goniometric measures

□ Other (please specify)

10. For the typical patient, how often do you assess passive limb ROM?

□ At least once per day on weekdays (Sun-Thu)

□ At least once per day, 7 days per week (ie including weekends)

□ Alternate days

□ Two to three times per week

□ Other (please specify)

11. For the typical patient, how long does assessment of his/her passive limb ROM take you?

□ < 5 minutes

□ 6-15 minutes

□ > 15 minutes

**SECTION 3: PHYSIOTHERAPY INTERVENTION**

**This section pertains to *intervention* only.**

12. Do you *routinely* treat *all* patients’ passive limb ROM irrespective of assessment findings?

□ Yes (please go to question 14) □ No

13. What criteria do you use to determine whether treatment of patients’ passive limb ROM is required?

Please tick all relevant boxes.

□ Reduction in limb ROM with respect to ‘normal’ parameters

□ Unilateral reduction in limb ROM in comparison to patient’s other side

□ Patient deemed at increased risk of loss of limb ROM (eg increased tone, burns,

pre-existing contracture)

□ Reason for admission (please give examples)

□ Other (please specify)

14. For the average ICU patient, what are your aims of treatment with respect to passive limb ROM?

Please *number* the boxes in order of importance (ie 1 = most important, 7 = least important).

□ Maintain joint ROM

□ Maintain soft tissue extensibility

□ Reduce loss of joint ROM

□ Reduce loss of soft tissue extensibility

□ Increase joint range of movement

□ Increase soft tissue extensibility

□ Preserve function

15. What treatment techniques/modalities do you use? Please *number* the boxes in order of frequency

of use (ie 1 = most commonly used, 7 = least commonly used).

□ Manually applied (ie by you) passive limb ROM

□ Orthoses/splints

□ Positioning regimen

□ Continuous passive motion machine

□ Neuromuscular electrical stimulation

□ Mobilisation (eg standing, walking)

□ Compression garments (eg gloves)

16. Does your aim of treatment affect what treatment techniques/modalities you use?

□ Yes (please specify how below) □ No

17. If you perform passive limb ROM as a treatment intervention, how often do you usually do it?

□ Twice daily weekdays (Sun-Thu)

□ Twice daily 7 days per week (including weekends)

□ Once daily weekdays (Sun-Thu)

□ Once daily 7 days per week (including weekends)

□ Alternate days

□ Other (please specify)

18. If you perform physiotherapist administered passive limb ROM as a treatment, what exercise

prescription do you usually use?

Number of repetitions per joint

Number of sets per joint

19. What parameter(s) do you use to limit physiotherapist administered passive limb ROM treatment?

Please tick all relevant boxes.

□ Onset of resistance

□ End of resistance

□ Onset of pain

□ Limit of pain

□ Other (please specify)

20. Do you reassess the effect of your passive limb ROM treatment? If yes, please indicate how you

reassess by ticking all relevant boxes.

□ Yes □ No

If Yes: □ Visual estimation

□ Goniometric measures

□ Other (please specify)

21. Do staff other than physiotherapists *ever* perform passive limb ROM with ICU patients? If yes,

please indicate which staff by ticking all relevant boxes.

□ Yes □ No (please go to question 24)

If Yes: □ Nursing staff

□ Physiotherapy assistants

□ Other (please specify)

22. On whose instruction do non-physiotherapy staff perform passive limb ROM with ICU patients?

Please tick all relevant boxes.

□ Medical staff

□ Physiotherapists

□ Nursing staff

□ Unit protocol

□ No instruction

23. For which ICU patients do non-physiotherapy staff perform passive limb ROM?

□ All patients

□ Patients with reduction in passive limb ROM with respect to ‘normal’ parameters

□ Physiotherapist deemed patient to be at increased risk of loss of passive limb ROM /

contracture (eg increased tone, burns, pre-existing contracture)

□ Other (please specify)

### SECTION 4: REFLECTION ON CURRENT PRACTICE

**In this section we want to find out the clinical rationale for your current physiotherapy service in ICU.**

24. In your ICU experience, how often do you encounter patients with loss of passive limb ROM / contracture?

□ Rarely ( < 0.1% admissions)

□ Uncommonly ( < 1% admissions)

□ Commonly ( 1-10% admissions)

□ Frequently ( > 10% admissions)

25. For those ICU patients who develop loss of passive limb ROM / contracture, how do you change your

management? Please tick all relevant boxes.

□ No change

□ Increase repetitions/sets of passive limb ROM

□ Increased frequency of passive limb ROM

□ Perform passive limb ROM into more resistance

□ Perform passive limb ROM into more pain

□ Increase usage of splints

□ Increase time in splints

□ Other (please specify)

26. For those ICU patients who develop loss of passive limb ROM / contracture, how much of a problem

do you think it is?

□ No problem (please go to question 29)

□ Minor

□ Moderate

□ Major

27. In what way(s) do you think it is a problem? Please *number* all boxes in order of importance (ie 1 =

most important, 6 = least important).

□ Patient cosmesis

□ Patient hygiene

□ Patient function

□ Patient quality of life

□ Increased hospital length of stay

□ Increased physiotherapy time required

28. On average, what percentage of your time in ICU would you spend doing passive limb ROM

treatments for ICU patients?

□ 0%

□ <25%

□ 25-50%

□ 51-75%

□ > 75%

29. Do you think your current practice with respect to passive limb ROM for ICU patients is effective?

□ Yes, always

□ Yes, mostly

□ Yes, occasionally

□ No

30. What factors / impairments do you think predispose ICU patients to loss of passive limb ROM /

contracture? Please tick all relevant boxes.

□ Burn injury

□ Orthopaedic injury

□ Neurological condition

□ Critical illness myoneuropathy

□ Increased tone

□ Weakness

□ Prolonged length of stay

□ Age of patient

□ Past history of musculoskeletal problems

□ Other (please list)

31. Please mark how much each of the following influence your physiotherapy practice with respect to

passive limb ROM for ICU patients. Please circle one number for each response.

1 2 3 4 5

No influence Very influential

Personal experience 1 2 3 4 5

Research findings 1 2 3 4 5

Advice from colleagues 1 2 3 4 5

Medical staff preferences 1 2 3 4 5

Resource/financial considerations 1 2 3 4 5

Established practice 1 2 3 4 5

Staffing numbers/caseload 1 2 3 4 5

Public/private patients 1 2 3 4 5

### SECTION 5: BACKGROUND ICU AND PERSONAL INFORMATION

This information will provide us with demographic data relating to you and your ICU. Although some data may seem irrelevant (e.g. age, gender) we need this information to be able to describe our study sample.

32. How many ICU beds are there in your hospital?

□ <15 beds

□ 16–30 beds

□ 31–50 beds

□ >50 beds

33. On average, how many patients are admitted to your ICU per year?

□ <50

□ 50-100

□ >100

34. What types of patients are admitted to your ICU? Please tick all appropriate boxes.

□ Medical

□ Surgical

□ Trauma

□ Burns

35. What, approximately, is the average length of stay for patients admitted to your ICU (days)?

36. What, approximately, is the average duration of ventilation for patients admitted to your ICU (hours)?

37. Please tell us your age

□ 20–30 years

□ 31–40 years

□ 41–50 years

□ >50 years

38. Gender □ Male □ Female?

39. How many years ago did you graduate with a Bachelor physiotherapy degree?

40. Do you have any postgraduate qualifications?

□ No (Please go to question 41)

□ DPT (Doctor of Physiotherapy)

□ Master's Degree in Physiotherapy

□ PhD (Doctor of Philosophy in Physiotherapy)

Other (please specify)

41. How many years of ICU physiotherapy experience do you have?

□ <5 years

□ 5-10 years

□ 11-15 years

□ >15 years

42. Has your ICU experience included personal participation in research?

□ No

□ Yes, as a researcher.

□ Yes, as a respondent only.
