# Supplementary material for: A national survey in United Arab Emirates on practice of passive range of motion by physiotherapists in intensive care unit
Source: PLoS One. 2021 Aug 20;16(8):e0256453. doi: 10.1371/journal.pone.0256453 (PMC8378748; doi:10.1371/journal.pone.0256453)
Supplement: Attachment — Submitted filename: Response to Reviewers.docx [file pone.0256453.s003.docx]

| **Dr. Walid Kamal Abdelbasset, Ph.D. , Academic Editor** | | | |
| --- | --- | --- | --- |
| Thank You for the corrections. We have tried our best to incorporate all the corrections. | | | |
| **SR. No.** | **CORRECTIONS** | **RESPONSE** | **PAGE No.** |
|  | **Journal Requirements** |  |  |
| 1. | Please ensure that your manuscript meets PLOS ONE's style requirements, including those for file naming. | Thank you for your comments, the manuscript has been modified to meet PLOS ONE’s requirements. | **-** |
| 2. | Please upload a copy of the questionnaire as a supplemental file. | The questionnaire has now been uploaded as a supplemental file titled “S1_Questionnaire “ | Supplemental file - S1_Questionnaire |
| 3. | We note that you have indicated that data from this study are available upon request. PLOS only allows data to be available upon request if there are legal or ethical restrictions on sharing data publicly. For information on unacceptable data access restrictions, please see <http://journals.plos.org/plosone/s/data-availability#loc-unacceptable-data-access-restrictions> | The data from this study has been made available and is attached as a supplemental file titled “S1_Data” | Supplemental file no. S2_Data |
| **Reviewer #1** | | | |
|  | Abstract | | |
| 1. | The background of the study is too long and also not justified the research gap. | Thank you for your comments. The background of the study has been revised and edited to be of reasonable length. Furthermore, the gap in research has been adequately justified. | 2 |
| 2. | The methods section is missing the study design, study setting, and the study duration. | The study design, setting and study duration have been added to the methods section. | 2 |
| 3. | The results component should consist of the correlation between the passive movement and its improvement in joint range of motion. | Thank you for the valuable suggestion. However, correlation analysis cannot be performed as actual values reflecting improvement in joint range of motion were not collected. | - |
| 4. | The conclusion is not drawn on the basis of the results drawn. | The conclusion has been modified to adequately reflect the results and discussion. | 2, 3 |
|  | Article | | |
| 1. | How come this study is differing from articles 9, 10, 11? | Thank you for requesting this clarification. The healthcare setting in the United Arab Emirates (U.A.E) especially pertaining to physiotherapy practice in unique. The role of physiotherapists varies widely based on hospital policies and protocols. Furthermore, in some hospitals, the role of the respiratory therapists and nursing professionals overlap with those of physiotherapists. This study aimed to explore the consistency in the practice patterns of licensed physiotherapists working in Intensive care units in the UAE with varied experience and diverse country of origin which is very typical as the country is largely made up of expats. | - |
| 2. | How come the UAE ethnicity and demographic characters are differed from England and Australia people? | The UAE is largely made up of an expatriate multicultural workforce even in the physiotherapy sector; this could possibly explain the differences seen. | - |
| 3. | Authors failed to find the research gap and its clinical significance. | The research gap with its clinical significance has been further highlighted. | 5 |
| 4. | Include the ethical committee name and its reference number. | This study has been approved by the Research Ethics Committee at the University of Sharjah. Reference number included. | 5 |
| 5. | Include the reliability and validity of the questionnaire used for this survey. | The content validity of the questionnaire has been added in the methods section. | 6 |
| 6. | Include the name of the questionnaire. | The name of the questionnaire has been included in the methods section. | 5 |
| 7. | Include the method used for finding the sample size? | The list of both private and governmental hospitals was obtained from the database of the Emirates physiotherapy society, UAE. Each hospital was contacted to ascertain the number of physiotherapists working in the Intensive Care Unit. From the hospitals that responded (both government and private) 54 physiotherapists were identified as working in ICUs. The response rate was approximately 61% and 33 physiotherapists completed the survey.  This explanation has been included in the manuscript.  Similar method as the survey done in Australia (Wiles L, Stiller K. Passive limb movements for patients in an intensive care unit: A survey of physiotherapy practice in Australia. J Crit Care. 2010;25(3):501–508.) was adopted. | 6 |
| 8. | Mention the statistical analysis performed for this survey. | Statistical analysis is included in the data analysis section and has been revised. | 7 |
| 9. | The results component should consist of the correlation between the passive movement and its improvement in joint range of motion. | Thank you for the valuable suggestion. However, correlation analysis cannot be performed as actual values reflecting improvement in joint range of motion were not collected. | - |
| 10. | Mention the role of age, gender, educational qualification, years of experience etc… in physical therapy intervention. | The practice patterns explored in the study may be influenced by the therapist’s qualifications, age, gender and years of experience Has been mentioned in the discussion. | 17 |
| 11. | The discussion part should define the mechanism, how passive movement improves patient’s symptoms in ICU with recent references. | Possible mechanisms of how passive movement might contribute to improvement in patients symptoms have been added to the discussion with recent references. | 16 |
| 12. | Overall, this study is not scientifically strong and also technically week for publishing. | The authors believe that with the modifications based on your valuable comments, the study could possibly be considered for publication. | - |
| **Reviewer #2** | | | |
| Abstract | | | |
| 1. | Line 33: Sample size extremely too small for a national survey. | Thank you for all your valuable suggestions. The number of physiotherapists practicing in Intensive care units in the UAE are relatively less.  Lists of both private and governmental hospitals was obtained from the database of the Emirates physiotherapy society, UAE. Each hospital was contacted to ascertain the number of physiotherapists working in the Intensive Care Unit. From the hospitals (both government and private) that responded 54 physiotherapists were identified as working in ICUs. The response rate was approximately 61% and 33 physiotherapists completed the survey.  This explanation has been included in the manuscript. | 6 |
| 2. | 51.6% is more than half and not almost half | This sentence has been removed from the abstract. | - |
| 3. | What assessment is being referred to? statement not clear. | Statement has been made clear. | 2 |
| Introduction | | | |
| 4. | Line 78: Effects should be changed to Benefits | The word “Effects” has been replaced by “Benefits” based on your valuable suggestion. | 4 |
| Materials and Methods | | | |
| 5. | Line 110: was made should be changed to was developed. | “Was made” has been changed to “was developed.” | 5 |
| 6. | Line 116: Reflection on their current practice is repeated. It is already written in line 113. | Repeated phrase removed. | - |
| 7. | Lines 122-125: What are the exclusion criteria. This statement on inclusion criteria is not clear. | The statement on inclusion and exclusion criteria has been revised and included in the Methods section. | 6 |
| 8. | Lines 126-127: Was required sample size not calculated? Were they from both private and government hospitals? | Lists of both private and governmental hospitals were obtained from the database of the Emirates physiotherapy society, UAE. Each hospital was contacted to ascertain the number of physiotherapists working in the Intensive Care Unit. From the hospitals (both government and private) that responded 54 physiotherapists were identified as working in ICUs. The response rate was approximately 61% and 33 physiotherapists completed the survey. This explanation has been included in the manuscript.  Similar method as the survey done in Australia (Wiles L, Stiller K. Passive limb movements for patients in an intensive care unit: A survey of physiotherapy practice in Australia. J Crit Care. 2010;25(3):501–508.) was adopted. | 6 |
|  | What physiotherapy Groups exactly? This has to be clearly stated. Would the national licensing body not be better? | The physiotherapy society “Emirates physiotherapy society” has been included revised manuscript. | 6 |
|  | Were they from both private and government hospitals? | The physiotherapists were from both government and private hospitals. This information has been added in the manuscript | 6 |
| Results | | | |
| 9. | Line 140: Are there a total 54 ICU physiotherapist with at least one year experience? | Yes, the list of physiotherapists working in ICU was obtained from Emirates physiotherapy society and all of them had atleast one year experience.  This has been clarified in the above section (Comment 8) and has been reflected in the revised manuscript. | 6 |
|  | Line 143: Table should be properly labelled as n (%), n, meaning frequency. | Table has been labelled appropriately. | 7,8 |
|  | The frequency data under age, gender and years of ICU experience do not add up to 33. | This mistake has been rectified and mentioned in the table. | 7,8 |
|  | The mean value for year of graduation says .3, I don’t know how that can be. | This mistake has been rectified and mentioned in the table. | 8 |
|  | The average duration of ICU stay should be clear if it was recorded in days, weeks or months. | Length of ICU stay was recorded in days and has been added in the table 1. | 8 |
|  | Under types of patients admitted to the ICU, Burns (the frequency and the percentage) was written twice. | Repeated words have been deleted. | 8 |
|  | What is GCC Region? Please be clear on that. | The region has been changed to be more specifically, the United Arab Emirates | 8 |
| 10. | Generally, be consistent with the number of decimal points. | Decimal points have been made consistent | Throughout the manuscript |
| 11. | Line 148: What is Blanket referral? Please be clear on that. | The meaning of “Blanket referral” has been clearly explained in the revised manuscript. | 8 |
| 12. | Line 151: Please include the standard deviation to the mean of 3.75 | Standard deviation has been included in the specified sentence. | 8 |
| 13. | Lines 166-168: It is good to clarify if the statement is per patient. | Statements have been clarified to reflect per patient. | 9 |
| 14. | Line 182: Tables 2 and 3. S should be added to table. | S added to the word table. | 10 |
| 15. | Line 190 and 194: Tables should be better labelled to indicate that these figures are all frequencies. The coding is not clear. | Tables have labelled appropriately. | 10, 11 |
| 16. | Line 204: Percentage of 37.5 cannot be referred to as majority. | Sentence has been revised. | 12 |
| 17. | Line 206 and 207: The percentage calculation of 26 (81.3%) or 26 (96.3%) is not correct. | Percentage calculations have been corrected. | 12 |
| 18. | Line 212: Frequency of 10 cannot be referred to as most of the respondents. Frequency of 10 cannot give a percentage of 55,6. | Sentence and percentage have been corrected. | 12 |
| 19. | Line 223: What percentage of the respondents reported contractures to be the major problem? | Percentage added in the sentence. | 12 |
| 20. | Line 238: Table should be better labelled to indicate that these figures are all frequencies. The coding should be made clearer. | Table has been labelled appropriately. | 13 |
| 21. | Line 249: According to figure 4, we had both neurological condition and burns, can this be made clearer? | Both neurological conditions and burns included in the sentence. | 14 |
| 22. | Line 260: Table should be better labelled to indicate that these figures are all frequencies. The coding should be made clearer | Table has been labelled appropriately. | 14 |
| 23. | Many of the percentage calculations are not correct. Some are lower, while some are higher. It is difficult to ascertain the actual sample size, 30 0r 33. Please this has to be clear, what exactly is the sample size? | Percentages throughout the manuscript have been corrected. | Throughout the manuscript |
| Discussion | | | |
| 24. | Line 269: What is Blanket referral? Can it be reworded? | The meaning of “Blanket referral” has been clearly explained. | 15 |
| 25. | Line 270: What other staff? Doctors? other physiotherapist? It has to be clearly stated. | Sentence has been revised. | 15 |
| 26. | Line 315: Who are senior physiotherapist? By age or by cadre? Are senior Physiotherapists not included at all in this study. | Sentence has been revised to clarify the definition of “senior physiotherapists” which depended on the number of years of experience. This study included both junior and senior physiotherapists. | 17 |
| 27. | Line 318-320: Could this be as a result of the mode of data collection? could it be that younger individuals generally have better reaction towards activities online? | Thank you for pointing out this possibility. Yes, online mode of data collection could possibly one of the reasons why more than half of of the respondents were juniors. | 17 |
| 28. | Line 321: I am worried that the sample size of 33 or 30 may not represent the perception of ICU physiotherapists in the UAE. | Although the number of respondents who participated in survey was small, this number could provide a representative sample considering that very few work in the Intensive Care Units in UAE. | - |
| 29. | What are the possible limitations to this study? | Limitations of the study have been added. | 17 |
| Acknowledgements | | | |
| 30. | Any acknowledgements? | Acknowledgements have been added. | 18 |
| Figures | | | |
| 31. | Can the bars on Fig 3 be better aligned? | The bars on Fig 3 have been aligned appropriately. | Fig3.tif |
| Raw Data | | | |
| 32. | Not available for review. | The raw data has been uploaded as a supplementary file. | Supplemental file no. S2_Data |
