# Supplementary material for: A national survey in United Arab Emirates on practice of passive range of motion by physiotherapists in intensive care unit
Source: PLoS One. 2021 Aug 20;16(8):e0256453. doi: 10.1371/journal.pone.0256453 (PMC8378748; doi:10.1371/journal.pone.0256453)
Supplement: Attachment — Submitted filename: Response to Reviewers.docx [file pone.0256453.s004.docx]

| **Dr. Walid Kamal Abdelbasset, Ph.D. , Academic Editor** | | | |
| --- | --- | --- | --- |
| Thank You for your comments. We have provided explanation for your valuable comments. | | | |
| **SR. No.** | **CORRECTIONS** | **RESPONSE** | **PAGE No.** |
| 1. | Five new authors have been added to the authors list. Each authors’ contribution should be explained. Please provide the authors’ contributions in line with ICMJE4 criteria. | All new authors contribution described accroding to ICMJE criteria:  -Acquisition, analysis, or interpretation of data for the work  -Drafting the work  -Final approval of the revision manuscript  -Agreement to be accountable for all aspects of the work in ensuring that questions related to the accuracy or integrity of any part of the work are appropriately investigated and resolved.  This project was part of research experience for students, and in the previous submission they were not included as they were still students. However, as they have now all graduated, they have been added based on their substantial contributions to the paper. This has been done accroding to Univeristy norms. Therefore, please consider addition of these authors to the list.  Their individual contribution has been highlighted as below:  **Conceptualization:** Gopala Krishna Alaparthi, Veena Raigangar, Kalyana Chakravarthy Bairapareddy, May Tamim  **Data curation:** Gopala Krishna Alaparthi, Veena Raigangar, Kalyana Chakravarthy Bairapareddy, Shamma Mohammad, Asma Alzarooni, Marah Atef, Rawan Abdulrahman, Sara Redha, Aisha Rashid  **Methodology:** Gopala Krishna Alaparthi, Veena Raigangar, Kalyana Chakravarthy Bairapareddy, Aishwarya Gatty, Shamma Mohammad, Asma Alzarooni, Marah Atef, Rawan Abdulrahman, Sara Redha, Aisha Rashid, May Tamim  **Resources:** Gopala Krishna Alaparthi, Veena Raigangar, Aishwarya Gatty  **Writing – original draft:** Gopala Krishna Alaparthi, Veena Raigangar, Kalyana Chakravarthy Bairapareddy, Aishwarya Gatty, Shamma Mohammad, Asma Alzarooni, Marah Atef, Rawan Abdulrahman, Sara Redha, Aisha Rashid, May Tamim  **Writing – review & editing:** Gopala Krishna Alaparthi, Veena Raigangar, Kalyana Chakravarthy Bairapareddy, Aishwarya Gatty | - |
| 2. | The major concern in the study is the small sample size compared with the study design. The authors have to explain how did they calculate the sample size and power of the study? | We only included the physiotherapists with atleast one year experience currently working in ICU. The sample size included in this study is small as there are only few physiotherapist treating patients in the ICUs in UAE.(we obtained a list of 54 physiotherapist out of which only 33 of them completed the survey) . Sample size was not calculated and we have included maximum number of samples that could be collected based on the eligibility criteria. This point-that the sample size in the study was small- has been included in the limitations of the study.  To obtain the total number of physiotherapist currently practising in ICUs, a list of both private and governmental hospitals was obtained from the database of the Emirates physiotherapy society, UAE. Each hospital was contacted through physical visits or phone calls to identify the number of physiotherapists working in the Intensive Care Unit. From the hospitals that responded (both government and private) 54 physiotherapists were identified as working in ICUs. The response rate was approximately 61% and only 33 physiotherapists completed the survey.  This explanation has been included in the manuscript.  Similar method as the survey done in Australia (Wiles L, Stiller K. Passive limb movements for patients in an intensive care unit: A survey of physiotherapy practice in Australia. J Crit Care. 2010;25(3):501–508.) was adopted. | 6,7 (Selection of the participants and recruitment)  17 (Limitations of the study-Discussion section) |
